# Exercise Intervention to Mitigate the Cardiovascular Sequence of Pregnancy Complications

**DOI:** 10.7759/cureus.75703

**Published:** 2024-12-14

**Authors:** Anurag Rawat, Kinnari Vyas

**Affiliations:** 1 Interventional Cardiology, Himalayan Institute of Medical Sciences, Dehradun, IND; 2 Plastic Surgery, Shri Guru Ram Rai Institute of Medical & Health Sciences, Dehradun, IND

**Keywords:** cardiovascular diseases, exercise, gestational diabetes, preeclampsia, pregnancy complications

## Abstract

Pregnancy issues such as gestational hypertension, preeclampsia, and gestational diabetes mellitus (GDM) are significant contributors to long-term cardiovascular diseases (CVDs) in women. Recent research has proved the impact of exercise on improving cardiovascular outcomes, particularly in women with pregnancy-related disorders. This review explores the outcomes of various exercise interventions on cardiovascular health in pregnant women. Among these, aerobic exercise has been widely studied, with results from observational studies and randomized controlled trials (RCTs) showing its positive outcomes on cardiovascular health in pregnant women, especially with complications. It has been found that regular aerobic exercise has been associated with reduced hypertension and improved endothelial function, particularly in women with a history of preeclampsia. Evidently, aerobic exercise results in better blood pressure regulation and enhanced vascular health that directly attends to the risk of cardiovascular diseases associated with pregnancy complications. Another form of exercise is resistance training, which despite being studied less, has shown potential benefits as well. Some advantages of resistance exercise have been found to improve muscle strength and overall enhancement in metabolic control. This is important, especially in women with GDM whereby improvement in insulin sensitivity reduces the overall risk of type 2 diabetes and future CVDs. Combined exercise that incorporates both aerobic and resistance elements has been known to offer the most comprehensive benefits. Various studies suggest that a combinatory approach maximizes the positive cardiovascular effects. Practicing women have experienced better overall heart health, with improved blood pressure regulation, enhanced endothelial function, and reduced metabolic risks. However, despite these findings, there are challenges such as small sample sizes and limited follow-up durations that hinder the generalizability of current research. Importantly, previous studies targeting exercise interventions for women experiencing complications during pregnancy have been limited in evidence by small sample sizes, short follow-ups, and lack of diversity. Such broader, more diverse populations were needed to reflect the various health risks and responses to exercise. Future research must include multi-center RCTs, diverse exercise regimens, and digital health tools for monitoring exercise adherence. This warrants future large-scale, multicenter trials that are necessary to establish more definitive evidence. Additionally, clinicians should consider including tailored exercise programs in care plans for women with pregnancy complications to mitigate long-term cardiovascular risks.

## Introduction and background

Pregnancy issues such as gestational hypertension, preeclampsia, and gestational diabetes mellitus (GDM) are significant health concerns that affect a considerable proportion of women globally.

These conditions not only pose immediate risks to maternal and fetal well-being but also have long-term implications for cardiovascular fitness of women. For instance, women with a background of preeclampsia have a two to fourfold elevated risk of getting hypertension, ischemic cardiac disease, stroke, and thromboembolic episodes later in life [1,2]. Similarly, GDM is linked to a heightened risk of type 2 diabetes, which is a well-established cardiovascular risk factor [3]. These connections underscore the need for effective interventions that can mitigate the long-term cardiovascular risks associated with pregnancy complications. Understanding the role of exercise in mitigating the cardiovascular risks associated with pregnancy complications is critical [4]. Exercise is a well-documented intervention for reducing cardiovascular risk factors in pregnant population, which is supported by several studies including a systematic review from Kramer and McDonald (2006) that regards all the types of exercises safe for the pregnant women and reported overall better pregnancy outcomes [5]. The decrease in blood pressure that comes with aerobic exercises is affected by improvements in cardiovascularity and flexibility of blood vessels. Resistance exercise naturally has a limited influence on blood pressure because the time varies within each session, consisting of short bursts of activity. During pregnancy, aerobic exercises such as walking and swimming are generally recommended for cardiovascular health and stamina, while resistance exercises should be avoided since most women will be heavier at the delivery stage. Rather, women should do lighter weights focusing more on form. The exercise guidelines during pregnancy recommend exercise at a moderate intensity while avoiding overheating, dehydration, and high-risk activities [4-6]. However, aerobic exercise significantly lowers the blood pressure in the at-risk gestational hypertensive women [6]. A RCT from Boparai et al. (2021) found that this benefit of aerobic exercise may be due to the improved microcirculatory dilatory capacity as evident from the hyperemia, but no significant change was observed in the endothelial functioning [7]. Meanwhile, the resistance exercise has been reported to have mixed outcomes when done alone. While some studies have reported benefits of resistance exercise alone on glycemic and blood pressure control, others found the effects insignificant [8]. It has been reported that combining aerobic and resistance exercises is effective in improving maternal cardiorespiratory fitness and reducing arterial stiffness, which is beneficial for cardiovascular health. This combination also shows a strong effect in reducing the risk of GDM and hypertensive disorders of pregnancy [9,10]. However, the specific impact of exercise on women with a history of pregnancy complications is less understood. Given the increasing prevalence of these complications and the associated long-term health risks, there is a pressing need to explore how exercise interventions could serve as a preventive strategy to enhance cardiovascular outcomes in this high-risk group [11]. The primary objective of this study is to synthesize existing evidence on the impact of exercise interventions on cardiovascular outcomes in women with a history of pregnancy complications. Specifically, this review aims to examine the physiological pathways linking pregnancy complications to increased cardiovascular risk. Additionally, we would explore the types of exercise interventions that have been studied in this population and their effectiveness. It includes analyzing the evidence from clinical studies regarding the cardiovascular benefits of exercise interventions. This will finally allow us to find the lacunae in the current research and propose future directions to advance understanding in this area.

## Review

Search strategy

In this study, the search strategy follows a systematic approach to addressing literature concerning exercise interventions and their effect on cardiovascular health especially addressing a population of women with pregnancy complications. This involves searching for databases like PubMed, Scopus, and Web of Science with the following keywords: pregnancy complications, cardiovascular, exercise interventions, gestational hypertension, preeclampsia, and gestational diabetes. Only studies that included some form of exercise intervention, be it aerobic, resistance, or both, and evaluated cardiovascular health in women with pregnancy-associated complications were accepted. RCTs, observational studies, and previous systematic reviews were the main types of studies included. Excluded studies are those that made no mention of cardiovascular issues, articles that had not undergone any form of academic peer review, and those with a lack of satisfactory data or authentic research work, were excluded from this review. The research team reviewed the title and abstract for relevance, and then, conducted a full-text review to assess if the study fit the inclusion criteria. Data from the studies that were included was extracted with emphasis on study design, study demographics, interventions, and outcomes, to identify gaps and measure the success of the interventions. This integrated strategy was developed in order to incorporate a sufficient quantity of evidence that will be used to assess how useful exercises are in reducing cardiovascular effects that are presented by pregnancy complications.

**Figure 1:**
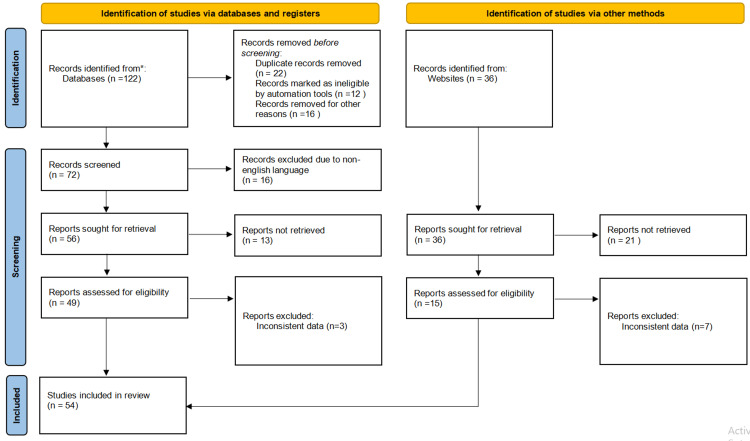
PRISMA flowchart * indicates databases such as PubMed, Scopus, and Web of Science. PRISMA: Preferred Reporting Items for Systematic reviews and Meta-Analyses

The Preferred Reporting Items for Systematic reviews and Meta-Analyses (PRISMA) flowchart (Figure 1) outlines the systematic process of study selection for a review. During the identification phase, 122 records were retrieved from databases, with 22 duplicates, 12 ineligible records flagged by automation tools, and 16 removed for other reasons, leaving 72 records for screening. An additional 36 records were identified through website searches, bringing the total to 108 records for further consideration. During the screening phase, 16 records were excluded due to being in non-English languages, and 13 reports could not be retrieved, resulting in 56 reports sought for retrieval from databases and 36 from websites. In the eligibility assessment stage, 49 database reports and 15 website reports were evaluated, leading to the exclusion of three records from databases and seven from websites due to inconsistent data. Finally, 54 studies were deemed eligible and included in the review. This flowchart provides a transparent account of the study selection process, ensuring reproducibility and reliability.

Physiological mechanisms linking pregnancy complications to cardiovascular risk

Pregnancy Complications and Cardiovascular Sequelae

Pregnancy complications such as gestational hypertension, preeclampsia, and GDM are linked to long-term cardiovascular risks through several physiological pathways. These complications can lead to persistent inflammation, endothelial dysfunction, and metabolic disturbances, all of which lead to the advancement of CVDs later in life. For example, preeclampsia is characterized by endothelial dysfunction, which can persist postpartum and predispose women to hypertension and atherosclerosis [12]. GDM, on the other hand, is associated with chronic hyperglycemia and insulin resistance, which are key drivers of type 2 diabetes and subsequent CVD [13].

Biological mechanisms

**Figure 2:**
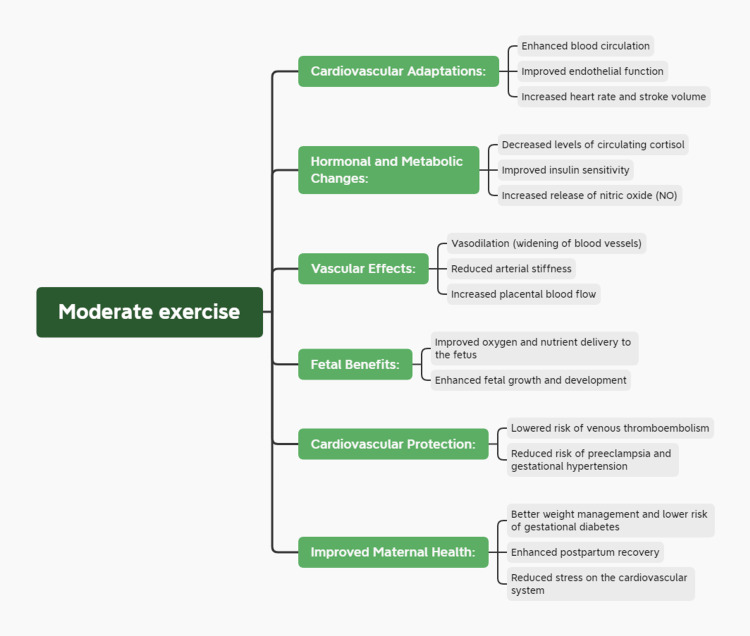
Physiological impacts of the moderate exercise on the overall pregnancy Source: Anurag Rawat

During pregnancy, among numerous other physiological changes, one prominent is the profound hemodynamic alteration (Figure 2) where there is a 50% elevation in cardiac functioning. Such a significant burden on the cardiovascular system of a mother provokes numerous factors that can potentially end up exacerbating cardiovascular complications [14]. Additionally, in the case of hypertensive disorders of pregnancies, that is, existence of gestational hypertension or preeclampsia or GDM, the risks of CVDs increase dramatically. These morbidities can potentially affect maternal energy metabolism which is often reported to be dysregulated in these conditions. Moreover, oxidative stress during pregnancy not only puts the maternal cardiovascular system at risk but also severely impacts the fetuses [15]. Due to this oxidative stress, numerous genetic and epigenetic alterations bring change in the gene expression resulting in adverse pregnancy outcomes [16]. One of those severe outcomes is atherosclerosis which is reported in the postpartum period [17]. All these changes lead to the higher expression of CVD biomarkers like glucose-insulin resistance, blood pressure fluctuations, and inflammatory markers during and even after pregnancy [18]. Therefore, the major biological mechanisms that link pregnancy complications to cardiovascular risk include inflammation, oxidative stress, and hormonal imbalances [19]. In preeclampsia, for instance, placental ischemia promotes the production of antiangiogenic factors such as soluble fms-like tyrosine kinase-1 (sFlt-1), which affects endothelial function and leads to hypertension. Additionally, oxidative stress resulting from elevated levels of reactive oxygen species (ROS) adds to endothelial damage and vascular failure [20]. In GDM, insulin resistance and chronic inflammation are central to pathophysiology, promoting atherosclerosis and increasing cardiovascular risk [21].

Role of exercise

Exercise has the potential to modulate these biological mechanisms by improving endothelial function, reducing inflammation, and enhancing insulin sensitivity. Aerobic exercise, for example, has been shown to increase nitric oxide bioavailability, which improves endothelial function and reduces blood pressure. Studies have demonstrated a percentage increase in nitric oxide availability of approximately 20-25% following moderate-to-vigorous aerobic exercise (e.g., cycling, jogging) [22]. This increase in nitric oxide helps to dilate blood vessels, thus improving blood flow and reducing hypertension. These effects are especially important in women with GDM, as they are at an elevated risk for developing cardiovascular complications later in life [20-23]. A study by Barakat et al. (2013) showed that resistance training in pregnant women with GDM led to a 16% reduction in insulin resistance [24]. Resistance training, on the other hand, can improve glucose metabolism and reduce insulin resistance, thereby decreasing the chances of getting type 2 diabetes in women with a background of GDM [23]. It has been well-established that obese women are particularly at risk of hypertensive disorders of pregnancies that eventually contribute significantly to cardiovascular diseases. Various studies have explored the role, impact, and importance of exercise during pregnancy to improve heart rate and maintain blood pressure. An RCT from Stutzman et al. (2010) reported that exercise during pregnancy not only reduced blood pressure but also resulted in the loss of parasympathetic tone particularly in overweight women [25]. Table 1 further provides the studies that have explored the mechanisms linking the pregnancy to cardiovascular complications.

**Table 1:** Physiological mechanisms linking pregnancy complications to cardiovascular risk

Study Objectives	Mechanism of Association	Results	References
To investigate how physiological changes during pregnancy could trigger or worsen cardiometabolic conditions	Significant hemodynamic shifts, such as a 50% increase in cardiac output, place stress on the maternal cardiovascular system during pregnancy	Cardiovascular issues arise in 1-4% of pregnancies, contributing to 16% of maternal fatalities	[14]
To analyze the metabolic and hemodynamic changes during pregnancy and their impact on long-term cardiovascular health	Failure to adapt to metabolic and hemodynamic changes can lead to hypertensive complications, gestational diabetes, and preterm birth	Pregnancy complications are linked to an elevated risk of future cardiovascular diseases and diabetes	[26]
To explore the pathophysiological connections between pre-pregnancy cardiovascular health, adverse pregnancy outcomes, and future cardiovascular disease	Unfavorable pregnancy outcomes may either indicate underlying cardiovascular risk or act as independent risk factors for future cardiovascular issues	Recent evidence supports the effectiveness of screening for subclinical cardiovascular disease in identifying high-risk postpartum women	[27]
To educate clinicians on the epidemiological evidence and guidelines for early intervention following complicated pregnancies	Hypertensive disorders in pregnancy contribute to vascular dysfunction and immune changes, which can lead to atherogenesis	The American Heart Association acknowledges pregnancy complications as independent risk factors for cardiovascular disease	[28]
To review normal cardiovascular physiology during pregnancy and its effects on maternal and fetal health	Systemic vasodilation and increased vascular elasticity are critical adaptations; inadequate adjustments can lead to complications such as preeclampsia	Pregnancy is associated with significant circulatory changes that can reveal underlying heart disease	[29]
To highlight the links between adverse pregnancy outcomes and future cardiovascular illness	Pregnancy complications may reveal existing high cardiovascular risk or directly contribute to long-term cardiovascular damage	Women with a history of adverse pregnancy outcomes have a higher likelihood of developing cardiometabolic risk factors and future cardiovascular disease	[30]
To examine the association of pregnancy complications with cardiovascular morbidity and mortality	Pregnancy complications are often preceded by subclinical vascular and metabolic disturbances	Pregnancy complications serve as important indicators of underlying high-risk cardiovascular conditions	[31]
To improve risk assessment for women with heart disease in order to provide appropriate obstetrical advice and care	Poor maternal functional status, myocardial dysfunction, and left heart obstruction are predictors of maternal cardiac complications	Pregnancy in women with heart disease is linked to significant cardiac and neonatal morbidity	[32]
To investigate the association of pregnancy complications with future cardiovascular disease risk and its contributing factors	Gestational diabetes and hypertensive disorders during pregnancy are linked to increased cardiovascular risk factors	Hypertensive disorders of pregnancy and gestational diabetes are independently associated with an elevated estimated 10-year cardiovascular disease risk	[33]
To examine the impact of advanced maternal age on cardiovascular adaptations during pregnancy and long-term health risks	Advanced maternal age may impair cardiovascular function during pregnancy, leading to suboptimal in utero environments	Advanced maternal age increases the risk of pregnancy complications and long-term cardiovascular disease for both the mother and offspring	[34]

Overview of exercise interventions

Types of Exercise

Exercise interventions in women with a background of pregnancy issues typically include aerobic exercise, resistance training, or a blend of both. Aerobic exercise, such as walking, swimming, or cycling, is known to improve cardiovascular fitness, lower blood pressure, and enhance insulin sensitivity. Resistance training, involving activities like weightlifting, helps in building muscle strength and improving metabolic health. Combined exercise programs, which integrate both aerobic and resistance training, may offer synergistic benefits by addressing multiple risk factors simultaneously [35,36].

Exercise Guidelines

Current exercise guidelines for pregnant women and those postpartum emphasize the significance of routine physical work to maintain cardiovascular health. The American College of Obstetricians and Gynecologists (ACOG) suggests at least 150 minutes of moderate-intensity aerobic activity per week during pregnancy and postpartum, with adjustments based on individual health status [AGOG 352020]. For women with a history of pregnancy complications, these guidelines are particularly relevant as exercise can help mitigate the increased cardiovascular risk associated with these conditions [37-41].

Target Populations

Exercise interventions are particularly beneficial for women with a background of preeclampsia, GDM, or gestational hypertension, as these populations are at a higher risk of developing cardiovascular diseases. For instance, women with a history of GDM benefit significantly from exercise interventions that enhance glucose metabolism and decrease the risk of type 2 diabetes [23,42,43]. Similarly, those who experienced preeclampsia may benefit from aerobic exercise programs that target endothelial dysfunction and hypertension [44-49]. Table 2 summarizes numerous studies that have explored different exercises to understand their impact on pregnancy and overall reduced risks of CVDs.

**Table 2:** Types of exercise interventions and their benefits GDM: Gestational diabetes mellitus; PE: pulmonary embolism

Study objectives	Type of Study	Type of Exercise	Outcome/Results	References
To evaluate the effects of exercise interventions for improving maternal and fetal outcomes in women with pre-existing diabetes	Systematic review	Various exercise programs	No RCTs identified; need for high-quality trials	[38]
To evaluate the effects of exercise interventions for improving maternal and fetal outcomes in women with gestational diabetes	Systematic review	Various exercise programs	Reduced fasting and postprandial blood glucose; insufficient data for other outcomes	[39]
To evaluate the implementation of a community-based exercise intervention during pregnancy	Descriptive, explorative	Moderate-intensity cardiovascular and strength training	High satisfaction, improved aerobic capacity, increased energy levels, no adverse effects	[40]
To evaluate the efficacy of an exercise intervention to prevent negative maternal and newborn health outcomes	Randomized controlled trial	Exercise-based intervention (3 times/week)	No significant differences in preterm birth, pre-eclampsia, gestational weight gain, gestational diabetes, birth weight, infant length, head circumference	[41]
To perform a systematic review and meta-analysis of the relationships between prenatal exercise and GDM, GH, and PE	Systematic review and meta-analysis	Various exercise programs	Exercise-only interventions reduced odds of GDM, GH, and PE	[10]
To discuss potential health benefits and harms of exercise during pregnancy	Clinical review	Various exercise programs	Reduction in Cesarean section rates, appropriate maternal and fetal weight gain, managing gestational diabetes; no reliable prevention of GDM, PE, or depression	[42]
To provide an update on the recent evidence concerning exercise during pregnancy	Systematic review	Various exercise programs	Higher cardiorespiratory fitness, prevention of urinary incontinence and low back pain, reduced symptoms of depression, gestational weight gain control	[43]
To evaluate the effectiveness of group exercise programs in improving pregnant women’s and newborns’ health outcomes	Systematic review	Group exercise programs including aerobic, resistance, pelvic floor training, stretching, relaxation	Improved health and fitness outcomes for women and newborns	[44]
To understand the evidence regarding maternal and offspring benefits of aerobic and/or resistance training during pregnancy	Systematic review	Aerobic and resistance training	Strong evidence for improved maternal cardiorespiratory fitness and prevention of urinary incontinence	[9]
To provide a comprehensive review of the risks and benefits and the prescription of physical exercise during pregnancy	Systematic review	Various exercise programs	Prevention of gestational diabetes, excessive gestational weight gain, hypertensive disorders, urinary incontinence, fetal macrosomia, anxiety, and depression	[42]

Evidence from clinical studies

Observational Studies

Several observational studies have examined the relationship between physical activity and cardiovascular outcomes in women with a history of pregnancy complications. For example, a large cohort study found that women who engaged in regular physical activity after a pregnancy complicated by preeclampsia had a significantly lower risk of developing hypertension and cardiovascular disease later in life [45]. Another study reported that postpartum physical activity reduced the risk of type 2 diabetes in women with a history of GDM [46-51].

Interventional Studies

Interventional studies, including RCTs, have provided more robust evidence of the efficacy of exercise interventions in this population. Moderate improvements have been observed in cardiovascular health due to the interventions in the meta-analysis with an effect size of 0.35 (95% CI: 0.10-0.60), which indicates a positive but modest impact on cardiovascular health. However, several of the cited studies suffer from certain methodological limitations. An important issue is the small sample size in most of the studies which tend to lower statistical power and the reliability of the findings derived from them. In addition, the nonrepresentation of study populations concerning age, ethnicity, and baseline health conditions makes it difficult to extrapolate the findings to larger populations that are more heterogeneous. The vast majority of the studies have included middle-aged adults from Western countries, who may not then well compare to the cardiovascular health outcomes in diverse, non-Western, or older populations. Such limitations indicate the importance of conducting larger, more representative trials to assess the effectiveness of interventions across demographic groups better [47-49]. Table 3 provides a summary of studies that have provided the benefits of exercise interventions during pregnancy.

**Table 3:** Summary of studies that explored the potential benefits of the exercise interventions during pregnancy

Type of Study	Objectives	Potential Benefit	Results	References
Clinical review	To discuss potential health benefits and harms of exercise during pregnancy	Reduction in Cesarean section rates, appropriate maternal and fetal weight gain, managing gestational diabetes	Exercise is safe for mother and fetus with several benefits, but evidence is inconsistent for preventing gestational diabetes, preeclampsia, or perinatal depression	[50]
Review of trials	To assess the effects of regular aerobic exercise on physical fitness, labor, delivery, and pregnancy outcomes	Improvement in physical fitness, body image	Regular aerobic exercise improves physical fitness and body image, but data are insufficient to infer important risks or benefits for mother or infant	[5]
Review of trials	To evaluate the effects of exercise interventions on maternal and fetal outcomes in women with pre-existing diabetes	Potential improvement in glycemic control, reduction in visceral adipose tissue and plasma triglycerides	No randomized controlled trials identified; need for high-quality trials to determine safe and effective exercise interventions	[38]
Committee opinion	To provide guidelines on exercise during pregnancy and postpartum	Decreased gestational diabetes, cesarean birth, operative vaginal delivery, postpartum recovery time, prevention of depressive disorders	Exercise is safe and beneficial for most women, with some modifications needed due to physiological changes	[51]
Literature review	To summarize research on changes in physical activity during pregnancy and its determinants	Mental and physical health benefits	Pregnant women are less active than non-pregnant women; higher education, income, and pre-pregnancy activity levels are predictors of higher exercise participation	[52]
Systematic review	To gather evidence on the risks and benefits of physical exercise during pregnancy	Prevention of gestational diabetes, excessive weight gain, hypertensive disorders, urinary incontinence, fetal macrosomia, lumbopelvic pain, anxiety, prenatal depression	Exercise is safe and beneficial, preventing several pregnancy-related disorders; compliance with guidelines is sufficient to achieve benefits	[42]
Systematic review	To assess the effects of exercise on pregnant women's quality of life	Improvement in quality of life	Group-based combined exercise and yoga or physical activity significantly improve quality of life; aerobic and resistance training have mixed effects	[53]
Systematic review	To evaluate the effectiveness of group exercise programs on health outcomes for pregnant women and newborns	Improvement in health, well-being, social support	Group exercise programs improve health and fitness outcomes for women and newborns	[44]
Systematic review	To update evidence on exercise during pregnancy, including effects on mother and fetus	Higher cardiorespiratory fitness, prevention of urinary incontinence and low back pain, reduced depression symptoms, gestational weight gain control	Exercise is beneficial for mother and fetus, not associated with risks for newborn, and can lead to long-term lifestyle changes	[43]

From numerous clinical studies, it is evident that physical activity interventions have shown positive outcomes, including increased physical activity levels, enhanced physiological adaptations, and decreased physical inactivity in women with a history of hypertensive disorders of pregnancy [52-54]. Additionally, it is also well reported that the combination of nutritional intervention and cardiovascular exercise starting six weeks postpartum significantly reduced arterial stiffness, a key cardiovascular risk factor, to levels comparable to women without pregnancy complications [55,56]. Other studies [57-59] have found that increased physical activity has been suggested to reduce the incidence of type 2 diabetes and improve cardiometabolic health in women with prior adverse pregnancy outcomes. As a result, women who maintain vigorous exercise throughout pregnancy have better long-term fitness, gain less weight, deposit less fat, and have a lower cardiovascular risk profile in the perimenopausal period compared to those who stop exercising. Despite these benefits, the current clinical guidelines often lack specific recommendations for managing cardiovascular risk factors in pregnant women, particularly those with a history of adverse pregnancy outcomes. Considering this there is a call to re-evaluate these guidelines to include exercise as a beneficial intervention [60,61].

Potential benefits of exercise interventions

Cardiovascular Benefits

Exercise interventions provide several direct cardiovascular benefits for women with a history of pregnancy complications. These include improved blood pressure control, better lipid profiles, and enhanced glucose metabolism, all of which contribute to reduced cardiovascular risk. Aerobic exercise, in particular, has been shown to lower systolic and diastolic blood pressure, which is crucial for women with a history of preeclampsia or gestational hypertension [48,62].

Psychosocial Benefits

Beyond the physical health benefits, exercise also offers significant psychosocial advantages, including improved mental health, quality of life (QoL), and overall patient satisfaction. Engaging in regular physical activity can reduce symptoms of depression and anxiety, which are common in postpartum women, especially those with a history of pregnancy complications [49]. Improved QoL and mental health outcomes further contribute to the overall well-being of these women, enhancing their ability to manage long-term health risks.

Safety and Feasibility

Exercise interventions for women with pregnancy complications are generally safe and feasible when tailored to the individual's health status and pregnancy history. Several studies have demonstrated that moderate-intensity exercise, under appropriate medical supervision, does not increase the risk of adverse pregnancy or postpartum outcomes [54].

**Figure 3:**
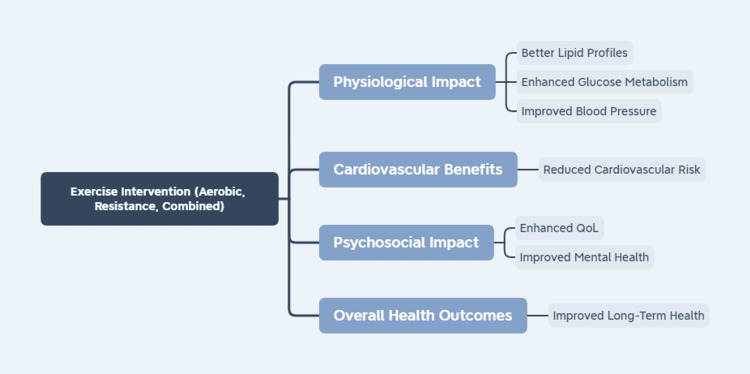
Flowchart of the pathways linking exercise to cardiovascular and psychosocial benefits Source: Anurag Rawat

Figure 3 shows the details of the pathway connecting exercise to cardiovascular and psychosocial benefits. 

Gaps in current research and future directions

Limitations of Existing Studies

Many of the existing studies on exercise interventions in women with a history of pregnancy complications are limited by small sample sizes, short follow-up periods, and a lack of diversity in study populations. Indeed, much of the current research that is done concentrates on white women but ignores black and other minority women who may be afflicted otherwise by different health challenges and responses to exercise. Not only is this another gap in demography, including things like socioeconomic status, cultural factors, or genetic differences, but it largely makes full assessment of outcomes in different populations impossible. These limitations hinder the generalizability of the findings and the ability to draw robust conclusions about the long-term benefits of exercise in this high-risk group [41].

Research Gaps

It requires more research with a broad and diverse population base in order for women from multiple ethnic, genetic, and socioeconomic backgrounds, who will have different risks and barriers associated with exercise, to be able to present themselves as having a more complete picture. The influence of these variables on exercise adherence and outcome should also be assessed. Different modalities of exercise, whether aerobic or resistance or a combined regimen, should be independently studied for their specific effect on cardiovascular risk factors such as blood pressure and cholesterol. Longitudinal exploratory studies are equally important for tracing the course of women during and after pregnancy and beyond, creating the potential for a better understanding of the effects of exercise interventions on long-term outcomes relating to the mother and her child [55,56].

Future Research Directions

In the future, there should be investigation in large, multi-center RCTs in a variety of populations seeing the efficacy of several types of exercises. This should include investigating the efficiency and feasibility of exercise regimens among women with histories of different pregnancy complications that also consider cultural and demographic differences. Research should involve the potential use of digital health interventions, like mobile health apps and telemedicine platforms, for the support and monitoring of exercise adherence. Successful instances of such digital tools, such as mHealth applications for the tracking of physical activities or telemedicine for remote consultation, shall be utilized to improve access to exercise programs in underserved communities in the future.

Clinical implications and recommendations

Implications for Practice

The findings from this review highlight the importance of integrating exercise interventions into the clinical care of women with a history of pregnancy complications. Healthcare providers should consider exercise as a key component of postpartum care, particularly for women at high risk of cardiovascular disease. Tailored exercise programs that consider the specific health status and pregnancy history of each woman can help mitigate long-term cardiovascular risks and improve overall health outcomes.

Recommendations for Clinicians

Exercise prescription should be aggressive for women who have previously suffered any pregnancy complications and give guidelines regarding the specifics, such as what type and how long to work out. Exercise programs should be individualized according to the particular complications of pregnancy, for example, gestational diabetes, pre-eclampsia, or the risk of preterm delivery. For example, a woman suffering from gestational diabetes would require personalized aerobic exercises to improve her insulin sensitivity, while a woman with a previous incidence of pre-eclampsia would probably benefit from resistance training due to a focus on cardiovascular health. It is also essential to offer support and resources, such as referrals to physical therapists or exercise programs, to ensure that these women can safely and effectively engage in regular physical activity. Clinicians should also consider the psychosocial benefits of exercise and encourage activities that promote mental well-being and improve QoL.

## Conclusions

This review has highlighted the significant cardiovascular risks associated with pregnancy complications and the potential of exercise interventions to mitigate these risks. The evidence suggests that regular physical activity, particularly aerobic and resistance exercise, can improve cardiovascular health, reduce the incidence of type 2 diabetes, and enhance mental well-being in women with a history of pregnancy complications. However, there is a need for more comprehensive research to fully understand the long-term benefits of exercise in this population and to develop tailored interventions that can be widely implemented in clinical practice. This analysis highlights how exercise, particularly aerobic and resistance training, reduces cardiovascular risks associated with pregnancy complications like gestational hypertension, preeclampsia, and GDM. It emphasizes the benefits of physical activity in improving blood pressure, endothelial function, and insulin sensitivity while acknowledging limitations in existing research due to small sample sizes and lack of diversity.

The present analysis recommends broader multicenter experiments aimed at determining long-term results as well as proper workout programs for varied categories of expectant mothers facing complications. From a clinical standpoint, this review suggests customizing exercise prescriptions for past pregnancies’ complications patients so as to reduce their cardiovascular risk levels and enhance their general wellness. This review highlights certain actionable recommendations for healthcare providers and researchers in order to promote moderate exercise during pregnancy including walking and swimming to pregnant women, emphasizing cardiovascular benefits. Furthermore, based on maternal health conditions and fitness levels a tailored exercise program must be recommended to ensure safety and maximize cardiovascular benefits. As a clinician, one important aspect would be to encourage prenatal and postnatal follow-ups to monitor cardiovascular health throughout pregnancy and postpartum to assess the long-term benefits of exercise on both mother and child. Meanwhile, researchers should explore biomarkers like nitric oxide levels, arterial stiffness, and endothelial function to understand the mechanisms of cardiovascular improvement through exercise. Special care and the development of exercise-based interventions must be ensured for high-risk groups that take care of pregnant women with preexisting cardiovascular risks or conditions like diabetes or obesity. In conclusion, by understanding the benefits of exercise, and prioritizing these strategies, healthcare providers as well as researchers can help optimize maternal and fetal cardiovascular health during pregnancy.
